# Retraction: Assessing the Contact Angle Between Dentin Treated With Irrigation and Calcium Hydroxide and Root Canal Sealers

**DOI:** 10.7759/cureus.r61

**Published:** 2022-09-29

**Authors:** Binila S Babu, Nithin Shetty, Nisha C, Noushin Faizal, Mrunalini Vaidya, Nishin K John

**Affiliations:** 1 Department of Conservative Dentistry and Endodontics, Al Azhar Dental College, Thodupuzha, IND; 2 Department of Conservative Dentistry and Endodontics, Ramaiah University of Applied Science, Bangalore, IND; 3 Department of Conservative Dentistry and Endodontics, Ministry of Interior, Doha, QAT; 4 Department of Conservative Dentistry and Endodontics, Dr GD Pol Foundation YMT Dental College, Mumbai, IND

This article has been retracted at the request of the authors due to a grouping error affecting the results and conclusion of the article. As stated by the authors, "Specimens G4, G5, and G6 were tested with bioceramic (BC) sealer rather than Tubli-Seal as stated in the article. Additionaly, they were subjected to irrigation for two weeks rather than the required four weeks. This also led to misinterpreted data by the authors. We sincerely regret the error." The Cureus editorial staff has reviewed the article and agreed to retract it per the authors' request.

